# Antiplatelet and anticoagulant agents in vitreoretinal surgery: a prospective multicenter study involving 804 patients

**DOI:** 10.1007/s00417-018-3968-y

**Published:** 2018-03-30

**Authors:** Andrzej Grzybowski, Piotr Kanclerz

**Affiliations:** 1 Department of Ophthalmology, University of Warmia and Mazury, Olsztyn, Poland; 2 Institute for Research in Ophthalmology, Foundation for Ophthalmology Development, ul. Gorczyczewskiego 2/3, 60-554 Poznan, Poland; 3 Department of Ophthalmology, Medical University of Gdańsk, Gdańsk, Poland

Dear Editor,

We have read the article by Meillon et al. [1] with great interest; however, we believe some discussion would be of benefit. The authors assessed the rate of hemorrhagic complications after vitreoretinal surgery and the influence of antithrombotic agents. This topic is particularly important, as the currently available literature is insufficient to decide whether to continue or discontinue anticoagulant and/or antiplatelet therapy during vitreoretinal surgery [2].

Chandra et al. [3] presented that the presence of dropped lens fragment is a risk factor for developing a suprachoroidal hemorrhage. We believe that some additional information from the study would be of importance. Were patients with dropped lens fragments included in the study, and was it a risk factor for hemorrhagic complications? Were phacovitrectomies performed in this study, as a combined procedure could present a different surgical time, risk for hypotony, and number of intraoperative maneuvers compared to a sole vitrectomy? Aphakia and pseudophakia were reported as risk factors for developing a suprachoroidal hemorrhage during vitrectomy in the past [4]. Thus, we would be interested in the patients’ lens status.

The majority of the evaluated surgeries were performed under peribulbar or retrobulbar anesthesia. Lid ecchymosis, as a hemorrhagic complication, was present in 1 out of 804 patients. The complications of local anesthesia in cataract surgery were meticulously investigated by Alhassan et al. [5] in the Cochrane Database for Systematic Reviews. The risk for lid haematoma was higher (7.3%) after retrobulbar block compared to peribulbar block (2.7%, RR 0.36, 95% CL 0.15 to 0.88). We would like to ask, what kind of needle was used for these blocks, and what was the exact definition of lid ecchymosis?

Finally, we would like to congratulate the authors for their excellent study in this very important field.
